# Expression of Salivary S100A7 Levels in Stage I Oral Submucous Fibrosis: A Clinical and Laboratory Study

**DOI:** 10.31557/APJCP.2020.21.4.1115

**Published:** 2020-04

**Authors:** Muhammad Arsalan Raffat, Naila Irum Hadi, Osama Alghamdi, Khulud Abdulrahman Al-Aali, Modhi Al Deeb, Tariq Abduljabbar, Fahim Vohra

**Affiliations:** 1 *Department of Oral Pathology, Baqai Dental College, Baqai Medical University, Karachi, *; 2 *Department of Pathology, Islamabad Medical and Dental College, Islamabad, Pakistan, *; 3 *Department of Oral and Maxillofacial Surgery, College of dentistry King Saud University, *; 4 *Department of Clinical Dental Sciences, College of Dentistry, Princess Nourah Bint Abdulrahman University, *; 5 *Department of Prosthetic Dental Science, College of Dentistry, King Saud University, *; 6 *Research Chair for Biological Research in Dental Health, Riyadh, Saudi Arabia. *

**Keywords:** Oral submucous fibrosis, saliva, S100A7, overexpression

## Abstract

**Background:**

Oral submucous fibrosis (OSF) is a chronic debilitating condition characterized by juxta-epithelial fibrosis. The main etiological agent associated with the high-risk precancerous condition is areca nut use. S100A7 is a member of the largest calcium-binding proteins exclusively found in vertebrates and are associated with the regulation of numerous intracellular and extracellular functions. The aim of this study was to investigate the expression of protein S100A7 in salivary samples of individuals with stage I OSF and healthy controls.

**Methods:**

This study included 63 participants, 30 of whom had OSF stage I and 33 healthy controls. Nonprobability quota sampling technique was utilized for recruitment of the study participants. A structured baseline questionnaire was used to collect demographic data. Saliva samples were collected by passive droll technique in a sterile container. Salivary levels of S100A7 were quantified by enzyme-linked immunosorbent assay. For the normality of the data Shapiro Wilk test was performed. Student t-test was commuted to evaluate the expression of S100A7 protein expression between both the study groups.

**Results:**

The mean salivary S100A7 value for stage I OSF group was 0.334 ng/ml, compared to 0.172 ng/ml for healthy controls. Student t-test reported a statistically significant difference, indicating higher levels of S100A7 in stage I OSF group than in healthy controls (p < 0.001). In the individual group analysis, a significant negative correlation was found between salivary S100A7 and duration of areca nut use (r = –0.45, p = 0.009) and gutka chewing (r = –0.20, p = 0.03), while a significant positive correlation was found between salivary S100A7 and mouth opening (r = 0.03, p = 0.04).

**Conclusions:**

Higher levels of S100A7 protein level was seen in stage I OSF group in comparison to the healthy individuals. Results of our study suggest that S100A7 could be used as a surrogate assessment to identify patients at risk of OSF development.

## Introduction

Oral submucous fibrosis (OSF) is a high-risk precancerous condition with a malignant transformation rate of about 7.6% with a follow-up of 17 years (Bansal et al., 2013; Arakeri et al., 2014). OSF is distinguished by severe lamina propria fibrosis and deeper connective tissue of the oral mucosa (Mehrotra et al., 2013). Approximately 2.5 million worldwide suffer from OSF, mostly in southern India. Areca nut used in various formulations has been identified by several studies as a significant risk factor associated with OSF (Hazarey et al., 2007). Pakistan is one of the countries in South-East Asia that have a broad habit of areca nut and smokeless chewable tobacco because their products are readily accessible that leads to increased OSF incidence in this country (Shah et al., 2009). A study conducted in the rural area of Sindh, Pakistan, recorded a higher (99%) incidence of OSF among consumers of areca nut and related products (Memon et al., 2015) In addition, studies in adolescents have recorded a relationship between OSF and areca nut use, indicating that 50 to 79.6% of users developed OSF (Ali et al., 2011; Maqsood et al., 2013).

The S100 proteins form the largest family of calcium-binding proteins, with approximately 25 members having a similar structure but encoded by separate genes (Donato et al., 2013; Wang et al., 2017). S100 proteins are found exclusively in vertebrates and were first isolated from brain samples in the early 1960s (Donato et al., 2013; Wang et al., 2017) These proteins play a vital role in intracellular, extracellular, and nuclear functions, including regulation of the cytoskeletal components, protein phosphorylation, enzymatic activity, calcium homeostasis, and p53 gene regulation (Donato et al., 2013).

S100A7 or psoriasin in the S100 family was initially isolated from skin (squamous epithelial cells) specimens of individuals with psoriasis (Madsen et al., 1991). In inflammatory or dysplastic lesions, altered S100A7 expression has been reported, and high levels of S100A7 in oral pre-cancerous lesions, showing that this protein can help diagnose these lesions (Zhou et al., 2008; Kaur et al., 2014). Trials using a non-invasive approach to examine salivary samples of patients with oral squamous cell carcinoma (OSCC) reported S100A7 overexpression, indicating that salivary S100A7 levels may assist in detecting early stages of this disease (Jou et al., 2014; Dey et al., 2015).

From the above results, we hypothesized that S100A7 is a possible biomarker for early detection of OSF and can help prevent this pre-cancerous condition from progressing to malignancy for the highly affected population (Memon et al., 2015). The aim of this clinical and laboratory study was to investigate the differential levels of S100A7 protein in saliva samples of patients with stage I OSF and matched healthy controls.

## Materials and Methods

*Ethical considerations*

This study was reviewed by the Ethics Research Committee of Ziauddin University (Reference code: 00190716MAOP). The protocol was presented with informed consent to all the enrolled patients before commencement.

*Power calculation*

Appropriate sample calculation was based on the percentage of OSF prevalence (Arakeri et al., 2014). With a precision of 2.5% and a confidence level of 95%, a total of 63 patients were selected through (non-probability) quota sampling, with 30 participants of stage I OSF and 33 healthy participants.

*Study patients and selection criteria*

This study consisted of 63 non-smoking participants divided into two groups: 30 patients with stage I OSF and 33 systemically and orally healthy participants. Without any gender predilection and with a history of areca nut and smokeless, chewable tobacco use were included in both groups. OSF patients previously treated with steroids or suffering from any chronic oral or systemic condition were not included.

*Research questionnaire*

The baseline structured questionnaire sheet was provided to all participants by a trained examiner. The questionnaire asked for details such as age, gender, occupation, ethnicity, habits (i.e., areca nut, betel quid, gutka use), and duration and frequency of habit. 

*Clinical examinations*

All participants were examined clinically. All OSF cases were diagnosed according to the clinical criteria proposed by Bose and Balan, using the stage I OSF intraoral assessment: (Bose and Balan, 2007) a) Unilateral or bilateral presence of thick fibrous bands in the buccal mucosae; b) opening of the mouth by estimating inter-incisal height c) restricted movement of the tongue; d) appearance of soft tissue in the tongue, buccal, palatal region and; e) degree of fibrosis in the soft palate.

*Saliva collection*

Unstimulated whole saliva (UWS) samples were taken from all patients using passive drool methods as defined elsewhere in a complete protocol (Akram et al., 2017). After the interview and clinical examination, 5 ml of UWS samples were collected from all participants in sterile falcon tubes (Fisher Scientific^®^, USA). The participants were comfortably seated with their head slightly bent forward and instructed to pool saliva in the mouth for 5 min. Participants pooled saliva in a wide funnel connected to a falcon tube placed in a cup filled with dry ice and transported to the laboratory immediately after collection. The samples were centrifuged at 1,792 x g for 10 min at 4°C and stored in multiple aliquots until further analysis at −80°C.

*ELISA for estimation of salivary S100A7*

The levels of S100A7 in saliva were measured using an enzyme-linked immunosorbent assay (ELISA) commercial kit (Abbexa Ltd, Cambridge Science Park, Cambridge, UK). Samples from all participants were quantified in triplicates. The minimum detection limit of for the kit was 0.156 ng/ml, with a sensitivity < 0.094 ng/ml. The assay procedure consisted of balancing all materials and prepared reagents to room temperature. Fifty microliters (μL) of the test and standard samples were added to the well plates. Antibody cocktail of 5 µL was added to the same well and incubated at room temperature for 60 min in a plate shaker (400 rpm). Later, all wells were washed three times with a wash buffer. Tetramethylbenzidine (TMB) substrate (100μL) was added to each well and incubated in the dark for 5 min in a plate shaker (400 rpm). The reaction was stopped by adding 100 µL of Stop solution to each well and the optical density absorbance was recorded at 450 nm in a microplate reader (Stat Fax 2100, USA) at 450 nm. Later, the concentration of S100A7 was calculated.

*Statistical analysis*

All data analyses were performed using specialized statistics software (SPSS v23 for Windows, IBM, Chicago, IL). Demographic data and salivary concentration S100A7 were reported as means and their standard deviations. Each variable was tested for normal distribution with Shapiro-Wilk test. Frequency distribution was analyzed using a descriptive analysis. A comparison of the S100A7 levels between the test and the controls was made using the student t-test. Correlations between the salivary S100A7 level and the independent variables were analyzed using the Pearson correlation test.

## Results

*General characteristics of the study participants*

The study included 63 participants (30 individuals with stage I OSF and 33 healthy controls). The percentage of male participants was 60.6% in the test group and 39.4% in the control group. The mean age for OSF and healthy controls was 27.3 years and 28.7 years, respectively. Among the 30 stage I OSF patients, 13 used areca nut (≈12.8 packets per day) and 17 used gutka (≈10.5 packets per day). Among the 33 healthy controls, 12 used areca nut (≈10.4 packets per day), 7 used gutka (≈10.5 packets per day), and 14 had no history of areca nut or gutka use. The mean duration of areca nut and gutka use among OSF patients was 8.5 and 6.7 years, respectively. The mean duration for healthy controls was 9.2 and 6.1 years, respectively. The mean mouth opening for patients with stage I OSF and healthy controls was 3.2 ± 0.68 cm and 3.9 ± 0.45 cm, respectively.

Among OSF patients, 20 had fibrosis of the right buccal mucosa, while 10 had fibrotic bands in the left buccal mucosa, 5 complained of restriction of the movement of the tongue movement, and 3 had slight fibrosis of the soft palate. A total of 16 patients reported a burning sensation in their mouth, while 14 patients had no burning sensation. 

*Mean concentration of salivary S100A7 among OSF patients and healthy controls*

**Figure 1.**
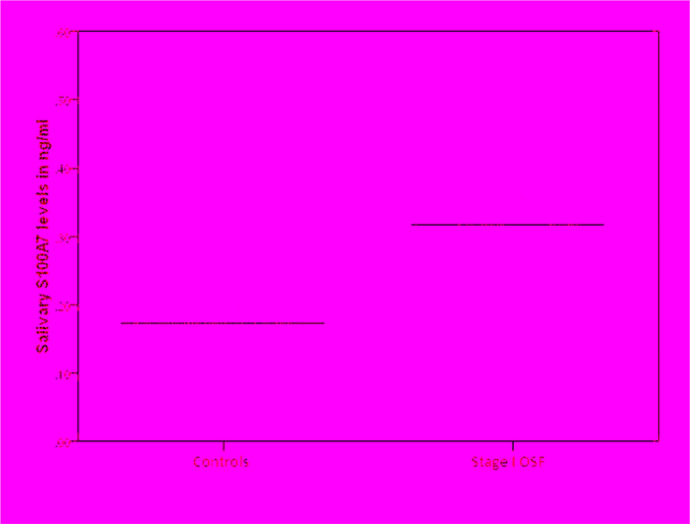
Scatter Plot Representing Salivary S100A7 Levels in Stage I OSF Patients and Healthy Controls

The mean salivary S100A7 levels for stage I OSF patients and healthy controls are shown in Table 1. The mean salivary flow rate was 0.44 ± 0.07 among stage I OSF patients and 0.54 ± 0.3 ml/min among healthy controls. The mean value of salivary S100A7 for the OSF group was 0.334 ± 0.075 ng/ml. The value for healthy controls was 0.172 ± 0.01 ng/ml. According to the student t-test, there was a statistically significant difference in S100A7 levels between the OSF group and the control group (p <0.001) (Figure 1).

*Pearson correlation analysis between salivary S100A7 levels and clinical variables*

The Pearson correlation coefficient was calculated to assess the correlations between salivary the S100A7 levels and the different clinical variables assessed in people with stage I OSF. There was a significant negative correlation between salivary S100A7 and duration of areca nut (r = –0.45, p = .009) and gutka use (r = –0.20, p = 0.03) when analyzed in each group. Additionally, there was a significant positive correlation between the opening of the mouth and the salivary S100A7 (r = 0.03, p = 0.04) (Table 2).

**Table 1:** Mean Salivary S100A7 Levels in Oral Submucous Fibrosis (OSF) Patients and Healthy Control

Variable	Group (n)	Mean ± SD (ng/ml)	Standard error of mean	95% CI	
				Lower bound	Upper bound
Salivary S100A7	Stage I OSF Patients (30)	0.334 ± 0.08*	0.016	0.305	0.362
	Healthy Controls (33)	0.172 ± 0.01	0.005	0.168	0.175

* *P*-value <0.001 by Independent t-test, which shows a significant difference between healthy controls and OSF patients.

**Table 2:** Pearson Correlation Analysis among Salivary S100A7, Habits, and Mouth Opening

	Correlation coefficient	*P*-value
Frequency of areca nut use		
Stage I OSF patients	-0.6	0.90
Healthy controls	0.36	0.22
Duration of areca nut use		
Stage I OSF patients	-0. 45	**0.009**
Healthy controls	0.01	0.92
Frequency of gutka use		
Stage I OSF patients	-0. 30	0.30
Healthy controls	-0. 25	0.39
Duration of gutka use		
Stage I OSF patients	-0. 20	**0.03**
Healthy controls	0.32	0.25
Mouth opening		
Stage I OSF patients	0.03	**0.04**
Healthy controls	0.05	0.85

## Discussion

In this study, it was assumed that salivary S100A7 levels were higher in individual with OSF than in healthy controls, and a correlation could be found between salivary S100A7 levels and different clinical indices. Our results showed that individual with stage I OSF expressed substantially more S100A7 than healthy controls. In fact, there was a significant positive correlation between salivary levels of S100A7 and mouth opening.

OSF demonstrates epithelial reduction and atrophy, inflammation in the juxta-epithelial section, absence of vascularization of the connective tissues, and weakened collagen activity, resulting in an increased inflammatory change (Pitiyage et al., 2011). S100A7 overexpression has been reported in several cancers, including OSCC (Zhou et al., 2008; Kesting et al., 2009; Raffat et al., 2018). Previous studies have shown that S100A7 binds directly to the advanced glycated end products receptors (RAGEs) and participate in proinflammation. RAGEs also suggest to help in the stimulation and activation of the nuclear factor kappa of activated B cells (NF-κB), a protein complex that promotes the production of proinflammatory cytokine (Wolf et al., 2011). S100A7 is also implicated to increase the levels of reactive oxygen species (ROS) in keratinocytes, creating a feedback loop where S100A7 is stimulated by ROS and intensified hypoxia (Vegfors et al., 2016). Our findings demonstrated higher levels of S100A7 in stage I OSF patients than in healthy controls. Our findings therefore suggest that S100A7 may be one of the major biomarkers of inflammatory changes in OSF.

Chewing substances containing areca nut and tobacco, such as betel quid and gutka, increases the risk of developing OSF (Niaz et al., 2017). Various organic alkaloids and flavonoids found in the areca nut stimulate collagen synthesis in fibroblasts, leading to a concomitant reduction of collagen degradation due to increased collagen stability and reduced collagenase activity (Arakeri et al., 2017). Previous data indicated that S100A7 had anti-fibrotic activity and reduced fibroblast proliferation in diseases associated with high collagen synthesis (Gauglitz et al., 2015). Therefore, it could be assumed that the resulted positive correlation between the two parameters (S100A7 and mouth opening) in stage I OSF patients illustrates a negative association between amount of S100A7 and increased production of collagen through fibroblasts in the oral mucosa of stage I OSF patients and hence the limited mouth opening.

Laboratory investigators regularly evaluate the presence of malignancy in different serum biomarkers. According to studies, the assessment of certain biomarker levels in saliva can also aid in detecting malignancies such as OSCC. Numerous research investigated salivary levels of S100A7, A8, A12, and S100P in OSCC patients (Jou et al., 2014; Dey et al., 2015). Such studies have suggested that S100 proteins are possible salivary biomarkers for human OSCC diagnosis and that a promising approach to detecting OSCC-related biomarkers that to determine the levels of these proteins in saliva. In clinical trials, saliva is the fluid of choice for routine evaluation of various protein. We tested the S100A7 levels of the saliva sample of stage I OSF patients and healthy controls. Detection of salivary levels S100A7 may have implications for precancerous oral lesions, such as OSF.

This research study has some limitations. It is a cross-sectional analysis and the character of the data does not enable us to understand the pathogenesis of OSF and its association at the molecular level with salivary S100A7 levels, which were evaluated at the same time as clinical examinations. In addition, we did not conduct microscopic evaluations to estimate the degree of fibrosis. Therefore, longitudinal studies can help to analyze the levels of S100A7 and correlate them with changes in soft tissues in OSF.

The classification of OSF according to various clinical and histopathological parameters is well-accepted (Bose and Balan, 2007). Nonetheless, the results of this study do not allow us to determine whether the severity of the disease and the malignant transformation are compatible with the salivary S100A7 levels. Observational studies are important for estimating salivary S100A7 levels in different disease stages. In this study, we relied solely on clinical examination to diagnose OSF. Invasive techniques such as “punch biopsy” allow pathologists to achieve a more accurate diagnosis and staging of OSF. However, such procedures have a significant impact on patient compliance.

This study shows that stage I OSF patients express more S100A7 in their saliva than the general population, suggesting that saliva measurement of S100A7 levels could be as responsive and accurate as serum measurements to distinguish healthy and precancerous states. Additional studies should however examine and compare the salivary S100A7 levels to those of serum and tissue samples, to confirm our results and to assess whether or not saliva is a reliable diagnostic fluid. We have found that S100A7 levels could be a diagnostic salivary protein to classify and recognize patients at risk of OSF. The sensitivity/specificity of S100A7 in saliva was not determined by this analysis. Thus, the threshold for diagnostic levels of S100A7 in OSF remains unknown. Preliminary findings are covered in this study, and further studies should be conducted to identify threshold levels of S100A7 in OSF.

In conclusion, in this study, we found that stage I OSF patients had higher levels of salivary S100A7 than healthy individuals. Our results suggest that S100A7 could be used as a surrogate assessment to identify patients at risk of OSF development. Nonetheless, further case-control studies based on disease severity should examine the S100A7 levels in advanced OSF stages to help better understand the role of this protein in disease progression.
